# Multimodal ultrasound features of breast cancers: correlation with molecular subtypes

**DOI:** 10.1186/s12880-023-00999-3

**Published:** 2023-04-17

**Authors:** Jun-Yan Zhu, Han-Lu He, Xiao-Chun Jiang, Hai-Wei Bao, Fen Chen

**Affiliations:** 1 Department of Ultrasound, Ruijin Hospital, Shanghai Jiao Tong University School of Medicine, Shanghai, China; 2 Department of Ultrasound, The First Affiliated Hospital of Zhejiang Chinese Medical University, Hangzhou, China; 3 Department of Ultrasound Medicine, The First Affiliated Hospital, Zhejiang University School of Medicine, Hangzhou, China

**Keywords:** Breast neoplasms, Molecular typing, Ultrasonography, Diagnostic imaging

## Abstract

**Objectives:**

To investigate whether multimodal intratumour and peritumour ultrasound features correlate with specific breast cancer molecular subtypes.

**Methods:**

From March to November 2021, a total of 85 patients with histologically proven breast cancer underwent B-mode, real-time elastography (RTE), colour Doppler flow imaging (CDFI) and contrast-enhanced ultrasound (CEUS). The time intensity curve (TIC) of CEUS was obtained, and the peak and time to peak (TTP) were analysed. Chi-square and binary logistic regression were used to analyse the connection between multimodal ultrasound imaging features and breast cancer molecular subtype.

**Results:**

Among 85 breast cancers, the subtypes were as follows: luminal A (36 cases, 42.4%), luminal B (20 cases, 23.5%), human epidermal growth factor receptor-2 positive (HER2+) (16 cases, 18.8%), and triple negative breast cancer (TNBC) (13 cases, 15.3%). Binary logistic regression models showed that RTE (P < 0.001) and CDFI (P = 0.036) were associated with the luminal A cancer subtype (C-index: 0.741), RTE (P = 0.016) and the peak ratio between intratumour and corpus mamma (P = 0.036) were related to the luminal B cancer subtype (C-index: 0.788). The peak ratio between peritumour and intratumour (P = 0.039) was related to the HER2 + cancer subtype (C-index: 0.859), and CDFI (P = 0.002) was associated with the TNBC subtype (C-index: 0.847).

**Conclusions:**

Multimodal ultrasound features could be powerful predictors of specific breast cancer molecular subtypes. The intra- and peritumour CEUS features play assignable roles in separating luminal B and HER2 + breast cancer subtypes.

## Introduction

Breast cancer is the most commonly diagnosed cancer and second leading cause of cancer death among women globally [1, 2]. Breast cancer is characterized by significant heterogeneity, leading to variable genetic, phenotypic and behavioural characteristics, clinical manifestations, and treatment reactions [3–8].

More recently, gene expression analysis with complementary DNA microarrays has been used to classify breast cancer into four molecular subtypes: luminal-A, luminal-B, human epidermal growth factor receptor-2 positive (HER2+), and TNBC [9, 10]. These subtypes have been demonstrated to represent prognostic and predictive information in breast cancers.

Ultrasound is a technology with the advantages of safety, noninvasiveness, and cost effectiveness that is commonly used for breast cancer diagnosis and screening [11]. To improve the diagnostic accuracy, we adopted multimodal information from B-mode, real-time elastography (RTE), colour Doppler flow images (CDFI) and contrast-enhanced ultrasound (CEUS) videos [12]. RTE can reflect the stiffness of the tissue [13]. CDFI can detect blood flow, which often increases in the tumour areas [14]. CEUS is a quantitative kinetic imaging method that can measure blood flow in breast tumours down to the level of the capillaries [15–19]. A cutting-edge technique for CEUS video quantification, time-intensity curve (TIC) analysis, extracts quantitative parameters of tumour blood perfusion [20–22]. These features obtained from multimodal ultrasound are associated with biological biomarkers and molecular subtypes and can help patients manage using precision treatment.

Regarding tumour growth and invasion, the microenvironment of tumours is recognized to play a critical role [23, 24], and peritumour tissue has been proven to provide useful information for diagnosis and prognosis prediction [25–29]. However, how peritumour tissue should be analysed has received relatively little attention [28].

In our study, we explored the correlation between breast lesion multimodal ultrasound features and specific breast cancer molecular subtypes to aid in the rapid diagnosis and early treatment of specific breast cancer molecular subtypes.

## Materials and methods

### Patient collection and breast cancer classification

The medical ethics committee of the First Affiliated Hospital of Zhejiang Chinese Medical University approved this retrospective study and complied with the Declaration of Helsinki. Informed consent was waived for this retrospective research.

From March to November 2021, 85 breast cancer patients from our hospital underwent ultrasound examinations (B-mode, RTE, CDFI, and CEUS), and images were recorded. We investigated the pathology reports of the patients by reviewing the clinical records.

The criteria for inclusion were as follows: (1) pathology confirmation of a newly diagnosed breast cancer; (2) initial diagnosis of unilateral invasive breast cancer with a single lesion; (3) no history of other organ cancers; (4) no neoadjuvant chemotherapy or endocrine therapy before surgery; (5) mass-forming breast lesions.

The criteria for exclusion were as follows: (1) breast cancer history; (2) multiple lesions in bilateral breast cancer; (3) ultrasound images of poor quality; (4) no postsurgical pathological reports; and (5) non-mass forming breast lesions.

Based on the Immunohistochemistry (IHC) results of oestrogen receptor (ER), progesterone receptor (PR), HER2, Ki-67, and fluorescence in situ hybridization (FISH), all breast cancers were divided into the following four molecular subtypes [9, 10]: (1) luminal A: ER and/or PR positive, and HER2-negative, and Ki67 < 14%; (2) luminal B: ER and/or PR positive, and HER2-negative, and Ki67 ≥ 14% or ER and/or PR positive and HER2-positive; (3) HER2+: HER2-positive, and ER and PR negative; and (4) TNBC: ER and PR negative, and HER2-negative.

### Ultrasound Examinations

All examinations were performed by an experienced sonographer (H. HL) with more than 15 years of breast technical and diagnosis experience so that the images were consistent. We employed a Techno MyLab Twice US system (Esaote, Genoa, Italy).

The LA523 probe with a frequency of 4–13 MHz was used for B-mode, RTE, and CDFI examinations. The protocol for breast scanning was as follows: The patient was placed in the supine position with the hands raised to fully expose the breast and armpits. The breast was scanned with continuous cross-section and longitudinal section. US images were all recorded on the largest transverse plane of the breast lesion.

RTE image acquisition was immediately performed and was conducted according to the World Federation for Ultrasound in Medicine & Biology guidelines for performing US elastography of the breast [46]. Briefly, the probe was vertically placed on the skin. An ultrasound probe was used to perform a light repetitive compression motion on the lesion. To obtain the best strain elastography, the pressure indicator on the screen was kept in green; that is, at least five of the seven pressure squares were displayed.

During CDFI examination, the blood flow in and around the lesions was observed, and a default equipment setting was implemented: a scale of 7 cm/s, a medium wall filter, and a pulse repetition frequency of 1.0 kHz.

Finally, we performed a CEUS examination using a 3–90 MHz linear transducer (LA522) probe. The machine was set as the default condition of breast CEUS. SonoVue (Bracco SpA, Milan, Italy) contrast agent was used. For contrast-tuned imaging, a bolus of 4.8 mL of contrast agent mixed with saline solution was injected via an antecubital vein, followed by aflush with 10 mL of 0.9% normal saline solution. Simultaneously, the dynamic pictures were recorded from the start of the injection and viewed for 120 s. The whole video was saved on the US machine for subsequent analyses.

### Ultrasound Image Analysis

Ultrasound images were assessed retrospectively by two sonographers (B. HW and J. XC) with more than 20 years of breast imaging and breast tumour diagnosis experience using a blinded study design, indicating that sonographers were not informed of the molecular subtype of each tumour throughout the analysis. The following features were evaluated: B-mode ultrasound image features (based on BI-RADS: shape, orientation, margin, echo pattern, posterior characteristic, and calcification), RTE score, and CDFI score. When the two sonographers reached opposing opinions, a chief physician sonographer (C. F, with more than 25 years of breast tumour diagnosis experience, recognized by the medical community) was consulted to make a definitive decision.

**Fig. 1:**
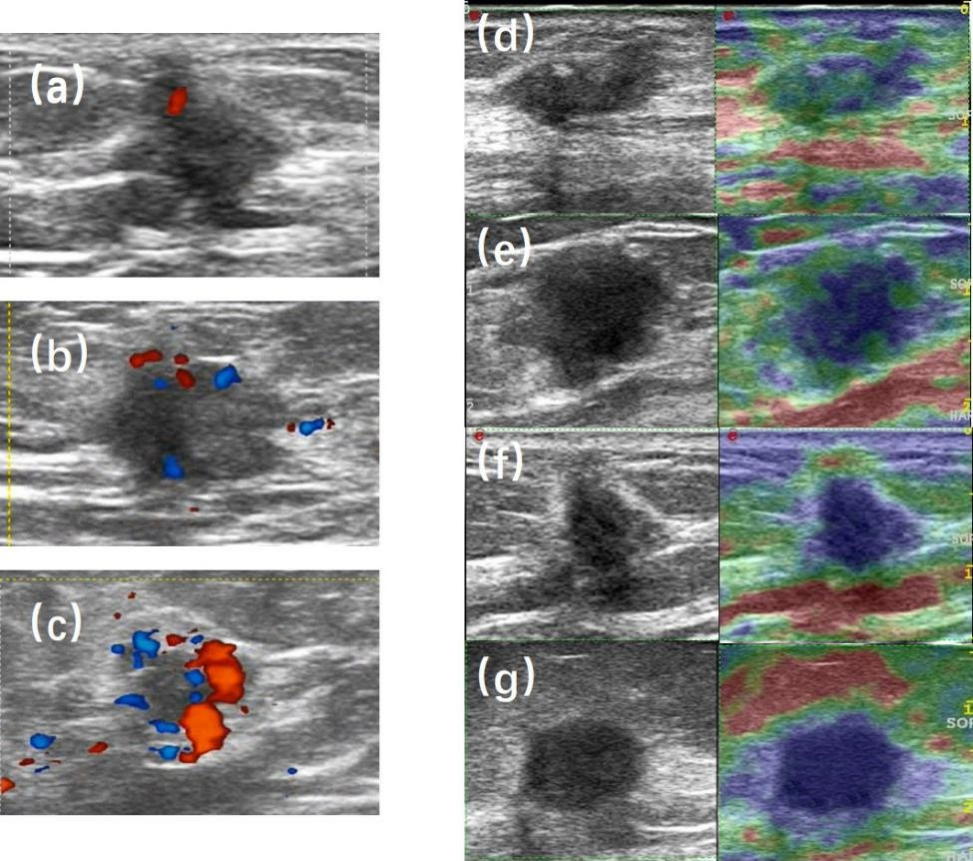
Examples of CDFI and RTE score. (a) CDFI grade 1; (b) CDFI grade 2; (c) CDFI grade 3; (d) RTE score of 2; (e) RTE score of 3; (f) RTE score of 4; (g) RTE score of 5. CDFI grade 0 and RTE score of 1 were not showed in our dataset.

RTE has a five-point scale. Itoh’s approach defined tumour stiffness as follows [13]: (1) score of 1: the entire lesion was evenly shaded in green; (2) score of 2: the hypoechoic lesion had a mosaic pattern of green and blue; (3) score of 3: the peripheral part of the lesion was green, and the central part was blue; (4) score of 4: the entire lesion was blue, but its surrounding area was not included; (5) score of 5: both the entire hypoechoic lesion and its surrounding area were blue (Fig. 1).

Adler’s approach defined tumour blood flow as follows [14]: (1) grade 0: no blood flow; (2) grade 1: minimum blood flow (1–2 dot-like signals or short-line-like signals); (3) grade 2: moderate blood flow (3–4 dot-like signals or 1 blood vessel longer than the lesion radius); and (4) grade 3: significant blood flow (3 or more blood vessels) (Fig. 1).

**Fig. 2:**
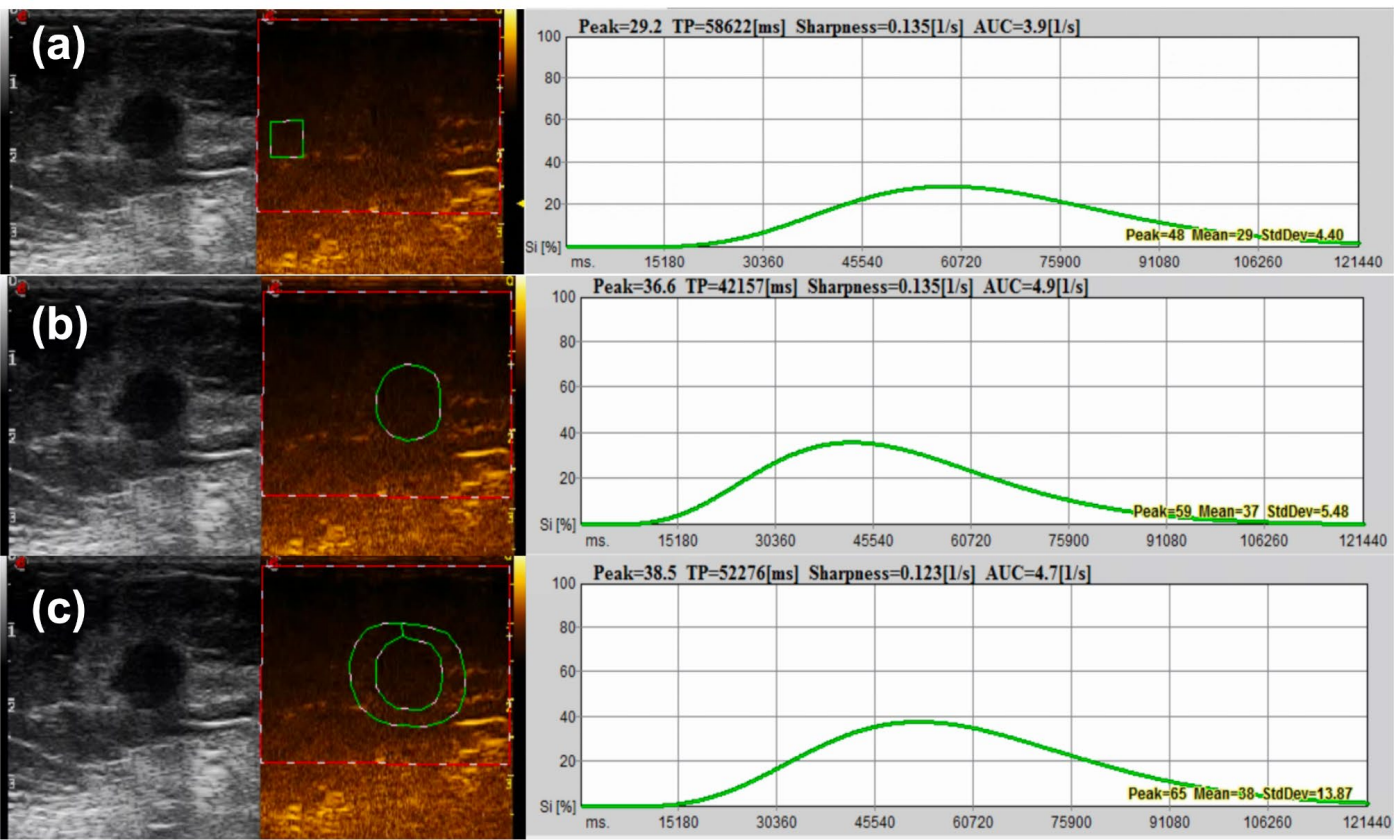
Contrast-enhanced ultrasound (CEUS) time intensity curve (TIC) analysis. The figure showed the Peak and Time to Peak (TTP). (a) ROI1: corpus mamma ROI; (b) ROI2: intratumour ROI; (c) ROI3: peritumour ROI.

The region of interest (ROI) in each CEUS was manually drawn along the tumour by a sonographer (Z. JY) who was blinded to the clinical and histological data of the patients, and the tissue 3–5 mm surrounding the tumour was defined as peritumour. Three ROIs were sketched in the CEUS of the same lesions—ROI1 (corpus mamma ROI), ROI2 (intratumour ROI) and ROI3 (peritumour ROI)—where the time-intensity curve (TIC) of the contrast transit was recorded by using QontraXt (Esaote, Genoa, Italy). Additionally, using analysis software, the quantitative parameters were computed. Two quantitative parameters of breast lesions on CEUS were observed: peak and time to peak (TTP) (Fig. 2).

Peak (%): the maximum intensity of the enhancing curve during the bolus given by the formula [(postcontrast signal—precontrast signal)/precontrast signal] ×100%.

TTP(s): the time from the appearance of the first microbubbles in the lesion to its maximum peak intensity.

### Data and statistical analysis

We focused on the relationship between ultrasound characteristics and specific subtypes of breast cancer. All the features were screened by chi-squared test and Fisher’s exact test and then were subjected to binary logistic analysis. Each subtype was subjected to a separate binary logistic regression analysis. Breast cancer molecular subtype was a binary variable in this study, with 1 indicating that a tumour is a subtype of interest, such as HER2+, and 0 indicating any other subtype. Binary logistic regression was performed for the four specific breast cancer molecular subtypes. The data were analysed by SPSS 26.0 (IBM, International Business Machines Corp., New York, US). P < 0.05 indicated a statistically significant difference.

## Results

### Baseline characteristics

Among 85 patients, 36 were luminal A, 20 were luminal B, 16 were HER2 + and 13 were TNBC. The mean participant age ± standard deviation was 52.9 years ± 9.2 (range, 30–77 years), and the mean tumour size was 2.33 ± 1.14 (range, 0.35–5.28 cm). The patient characteristics and tumour histopathologic features are summarized in Table 1.

**Table 1:** Patient and tumour characteristics

Characteristics	n (%)
**Age (y)**	
Mean ± standard deviation	52.9 ± 9.2
Median (Range)	53 (30–77)
**Tumour size (cm, ± SD)**	
Mean ± standard deviation	2.33 ± 1.14
Median (Range)	1.97 (0.35–5.28)
**Histopathology**	
Invasive ductal cancer	77 (90.5)
Invasive lobular cancer	5 (6.0)
Other*	3 (3.5)
**Histologic grade**	
Grade 1	15 (17.6)
Grade 2	26 (30.6)
Grade 3	44 (51.8)
**Estrogen receptor**	
Negative	29 (34.1)
Positive	56 (65.9)
**Progesterone receptor**	
Negative	36 (42.4)
Positive	49 (57.6)
**HER2**	
Negative	54 (63.5)
Positive	31 (36.5)
**Ki-67**	
High ( > = 14%)	57 (67.1)
Low (< 14%)	28 (32.9)
**Molecular subtype**	
Luminal A	36 (42.4)
Luminal B	20 (23.5)
HER2+	16 (18.8)
TNBC	13 (15.3)

* Invasive tubular, mucinous.

### Correlation between the B-mode、 RTE、 CDFI ultrasound features and specific breast cancer molecular subtypes

The luminal A cancer subtype was significantly correlated with tumour nonparallel orientation (p = 0.014), a spiculated margin (p = 0.022), no calcification (p = 0.004), high stiffness (p < 0.001), and a weak blood flow signal (p = 0.004).

The luminal B cancer subtype was significantly associated with tumour low stiffness (p = 0.003).

The HER2 + cancer subtype significantly corresponded with the tumour microlobulated margins (p = 0.024), isoechoic echo patterns (p = 0.003), and a rich blood flow signal (p = 0.039).

The TNBC subtype was significantly associated with a rich blood flow signal of the tumour (p = 0.007).

The tumour ultrasound shape and posterior features did not significantly correspond to any molecular subtypes. The results are shown in Table 2.

**Table 2:** The tumour B-mode、RTE、CDFI ultrasound features per molecular subtype

Molecular Subtype	Total [n = 85]	Luminal A [n = 36] VS. Other Types		Luminal B [n = 20] VS. Other Types		HER2+ [n = 16] VS. Other Types		TNBC [n = 13] VS. Other Types	
	N	Luminal A	p	Luminal B	p	HER2	p	TNBC	p
**Tumor size (cm, Mean ± SD)**	2.33 ± 1.14	1.72 ± 0.73		2.61 ± 1.23		3.09 ± 1.19		2.67 ± 1.14	
**Shape**			0.516		0.730		0.115		0.362
Oval/Round	11(12.9)	6(16.7)		2(10.0)		0(0.0)		3(23.1)	
Irregular	74(87.1)	30(83.3)		18(90.0)		16(100)		10(76.9)	
**Orientation**			0.014		1.000		0.111		0.198
Parallel	73(85.9)	27(75.0)		17(85.0)		16(100)		13(100)	
Non-parallel	12(14.1)	9(25.0)		3(15.0)		0(0.0)		0(0.0)	
**Margin**			0.022		0.330		0.024		0.682
Circumscribed	0(0.0)	0(0.0)		0(0.0)		0(0.0)		0(0.0)	
Microlobulated	8(9.4)	1(2.8)		1(5.0)		5(31.2)		1(7.7)	
Spiculated	24(28.2)	16(44.4)		3(15.0)		3(18.8)		2(15.4)	
Angular	41(48.3)	15(41.7)		12(60.0)		6(37.5)		8(61.5)	
Indistinct	12(14.1)	4(11.1)		4(20.0)		2(12.5)		2(15.4)	
**Echo pattern**			0.105		1.000		0.003		1.000
Hyperechoic	3(3.5)	2(5.6)		0(0.0)		1(6.3)		0(0.0)	
Isoechoic	5(5.9)	0(0.0)		1(5.0)		4(25.0)		0(0.0)	
Hypoechoic	77(90.6)	34(94.4)		19(95.0)		11(68.7)		13(100)	
**Posterior features**			0.330		1.000		0.338		0.115
No posterior features	49(57.6)	21(58.3)		12(60.0)		8(50.0)		8(61.5)	
Combined pattern	6(7.1)	1(2.8)		1(5.0)		2(12.5)		2(15.4)	
Enhancement	2(2.4)	0(0.0)		0(0.0)		1(6.3)		1(7.7)	
Shadowing	28(32.9)	14(38.9)		7(35.0)		5(31.2)		2(15.4)	
**Calcifications**			0.004		0.051		0.180		1.000
No	28(32.9)	18(50.0)		3(15.0)		3(18.8)		4(30.8)	
Yes	57(67.1)	18(50.0)		17(85.0)		13(81.2)		9(69.2)	
**RTE**			0.001		0.003		0.280		0.339
1	0(0.0)	0(0.0)		0(0.0)		0(0.0)		0(0.0)	
2	44(51.8)	8(22.2)		13(65.0)		12(75.0)		11(84.6)	
3	6(7.1)	1(2.8)		4(20.0)		1(6.3)		0(0.0)	
4	19(22.3)	13(36.1)		3(15.0)		2(12.5)		1(7.7)	
5	16(18.8)	14(38.9)		0(0.0)		1(6.3)		1(7.7)	
**CDFI**			0.004		0.097		0.039		0.007
0	0(0.0)	0(0.0)		0(0.0)		0(0.0)		0(0.0)	
1	11(13.0)	9(25.0)		2(10.0)		0(0.0)		0(0.0)	
2	42(49.4)	19(52.8)		14(70.0)		6(37.5)		3(23.1)	
3	32(37.6)	8(22.2)		4(20.0)		10(62.5)		10(76.9)	

### Correlation between the CEUS features and specific breast cancer molecular subtypes

Regarding CEUS features, in our study, we used the peak ratio among corpus mamma (ROI 1), intratumour (ROI 2) and peritumour (ROI 3) for analysis. The results are shown in Table 3.

**Table 3:** The tumour CEUS features per molecular subtype

Molecular Subtype	Total [n = 85]	Luminal A [n = 36] VS. Other Types		Luminal B [n = 20] VS. Other Types		HER2+ [n = 16] VS. Other Types		TNBC [n = 13] VS. Other Types	
	N	Luminal A	p	Luminal B	p	HER2	p	TNBC	p
**Peak ROI2/ Peak ROI1**			0.694		0.050		1.000		0.588
<=1	7 (8.2)	2(5.6)		4(20.0)		1(6.3)		0(0.0)	
> 1	78 (91.8)	34(94.4)		16(80.0)		15(93.8)		13(100)	
**Peak ROI3/ Peak ROI1**			0.571		0.558		1.000		1.000
<=1	3 (3.5)	2(5.6)		1(5.0)		0(0.0)		0(0.0)	
> 1	82 (96.5)	34(94.4)		19(95.0)		16(100)		13(100)	
**Peak ROI3/ Peak ROI2**			0.843		0.620		0.014		0.009
<=1	25 (29.4)	11(30.6)		5(25.0)		9(56.2)		0(0.0)	
> 1	60 (70.6)	25(69.4)		15(75.0)		7(43.8)		13(100)	

The peak ROI2/peak ROI1 was significantly associated with the luminal B cancer subtype (p = 0.050). Compared with the peak of corpus mamma, low enhancement of the intratumour was associated with luminal B.

Peak ROI3/Peak ROI2 was significantly correlated with HER2+ (p = 0.014) and TNBC (p = 0.009) subtypes. Compared with the intratumour peak, the low peritumour enhancement is associated with HER2+, and the high peritumour enhancement is associated with TNBC.

The analysis of Peak ROI3/Peak ROI1 and TTP did not significantly correspond with any molecular subtypes.

### Binary logistic regression analysis of specific breast cancer molecular subtypes

The binary logistic regression analysis results are summarized in Table 4.

**Table 4:** Binary logistics regression analysis of molecular subtypes with ultrasound feature

Molecular Subtypes	Feature	Sig.	OR	95% CI
Luminal A vs. Other Types	Orientation	0.409	0.486	0.088–2.693
	Margin	0.374	0.698	0.317–1.541
	Calcification	0.360	1.834	0.501–6.712
	RTE	< 0.001	0.322	0.188–0.550
	CDFI	0.036	1.1066	1.066–6.987
Luminal B vs. Other Types	RTE	0.016	1.920	1.127–3.272
	Peak ROI2/ Peak ROI1	0.036	6.654	1.128–39.272
HER2 + vs. Other Types	Margin	0.172	1.639	0.806–3.331
	Echo pattern	0.055	3.017	0.976–9.333
	CDFI	0.052	0.348	0.120–1.009
	Peak ROI3/ Peak ROI2	0.039	3.796	1.067–13.500
TNBC vs. Other Types	CDFI	0.002	0.106	0.025–0.449
	Peak ROI3/ Peak ROI2	0.998	0.000	0.000

Binary logistic regression showed that RTE (P < 0.001) and CDFI (p = 0.036) were predictive of the luminal A cancer subtype (C-index: 0.741). RTE (P = 0.016) and the peak ratio between intratumour and corpus mamma (P = 0.036) were predictive of the luminal B cancer subtype (C-index: 0.788). The peak ratio between peritumour and intratumour (P = 0.039) was independently predictive of the HER2 + subtype (C-index: 0.859); CDFI (P = 0.002) demonstrated excellent discrimination for predicting the subtype of TNBC (C-index: 0.847).

## Discussion

Breast cancer has traditionally been regarded as a heterogeneous disease [30, 31]. The identification of human breast cancer subtype-specific molecular features has substantial implications for clinical treatment options, disease progression, and ultimately patient prognosis [32, 33]. Specific subtypes of breast cancer differ not only in microscopic features but also in imaging analyses. Our study demonstrated associations between multimodal ultrasound imaging features and specific breast cancer molecular subtypes.

The microenvironment of breast cancer, measured indirectly in ultrasound images by its stiffness, is an additional essential feature explored in the medical literature [34]. Yoo et al. found that tumour hypoxia may be the root cause of tumour stiffness and found that the stiffness of tumours is higher in triple-negative or HER2 + cancer than in luminal-type cancer [35]. However, we found that RTE could predict luminal subtypes, high stiffness of breast tumours is related to luminal A, and low stiffness is related to luminal B. The Luminal A subtype of cancers is associated with a relatively favorable prognosis, and most are low-grade tumours. High stiffness is more likely in low-grade breast cancer that are associated with desmoplastic reactions [36, 37]. Some studies have reported that the combination of CDFI with B-mode ultrasound can improve the diagnosis of breast cancer [38]–40]. Similar to other studies, we found that tumours with an insufficient blood supply are related to luminal A, while those with an abundant blood supply are related to TNBC. TNBCs are associated with aggressive biological characteristics, poor clinical outcomes and limited therapeutic methods. Hypervascularity is associated with the rapidly aggressive proliferating pattern of TNBCs, and hypovascularity of Luminal A subtype is related to its low-grade [41, 42].

CEUS has demonstrated excellent effectiveness in detecting both large and small vascularities, indicating arterial perfusion in and around breast cancer tissues [43]. CEUS characteristics are beneficial for discriminating benign and malignant breast tumours and predicting breast cancer prognostic factor expression [44, 45], which is generally accepted. We further explored the role of CEUS and found that it is also valuable in molecular typing. We found that the luminal B cancer subtype is associated with low enhancement of the intratumour (relative to the corpus mamma), and high enhancement of the intratumour (relative to the peritumour) independently predicts the HER2 + cancer subtype, the enhancement pattern may be related to its different components of intratumour and peritumour. All TNBC showed high peritumour enhancement (relative to the intratumour), might be associated with internal necrosis of tumour formed by the rapid growth, so that less enhancement intratumour.

Metabolism and blood flow are both fundamentally important for normal cell survival and tissue viability. However, in tumour cells, both the vascular supply and energy metabolism are disorganized [46, 47]. Tumour blood flow differs across breast cancer tumour subtypes. Previous studies have demonstrated that tumour blood flow and metabolism differ across breast cancer tumour subtypes, a finding that is congruent with the molecular heterogeneity across tumour types identified by gene profiling [48, 49].

Our study had the following limitations: we based our analysis on breast tumours from a single centre, and it was a retrospective analysis. Furthermore, multiple data could help make more accurate models that can predict the molecular subtype of breast cancer.

## Conclusions

Our investigation suggested that high stiffness and insufficient blood supply of breast tumours is related to luminal A. Low stiffness of tumour and low enhancement of intratumour relative to the corpus mamma is related to the Luminal B cancer subtype. High intratumour enhancement compared with peritumour enhancement is related to the HER2 + cancer subtype. Tumour with an abundant blood supply and high peritumour enhancement are related to TNBC. These multimodal ultrasound features, especially intra- and peritumour CEUS features, may help noninvasively predict specific subtypes.

## Data Availability

The datasets used or analysed during the current study are available from the corresponding author on reasonable request.

## List of abbreviations

RTE: Real-Time Elastography
CDFI: Color Doppler Flow Images
CEUS: Contrast Enhanced Ultrasound
TIC: Time Intensity Curve
TNBC: Triple Negative Breast Cancer
IHC: Immunohistochemistry
FISH: Fluorescence in situ Hybridization
ROI: Region of Interest
TTP: Time to Peak

## References

[1] Siegel RL, Miller KD, Jemal A (2018). Cancer statistics. CA Cancer J Clin.

[2] Schick J, Ritchie RP, Restini C (2021). Breast Cancer therapeutics and biomarkers: past, Present, and future approaches. Breast Cancer (Auckl).

[3] Zardavas D, Irrthum A, Swanton C, Piccart M (2015). Clinical management of breast cancer heterogeneity. Nat Rev Clin Oncol.

[4] Chew NJ, Lim Kam Sian TCC, Nguyen EV, Shin SY, Yang J, Hui MN (2021). Evaluation of FGFR targeting in breast cancer through interrogation of patient-derived models. Breast Cancer Res.

[5] Tashireva LA, Savelieva OE, Grigoryeva ES, Nikitin YV, Denisov EV, Vtorushin SV (2021). Heterogeneous manifestations of epithelial-mesenchymal plasticity of circulating Tumor cells in breast Cancer patients. Int J Mol Sci.

[6] Haynes B, Sarma A, Nangia-Makker P, Shekhar MP (2017). Breast cancer complexity: implications of intratumoral heterogeneity in clinical management. Cancer Metastasis Rev.

[7] Martelotto LG, Ng CK, Piscuoglio S, Weigelt B, Reis-Filho JS (2014). Breast cancer intra-tumor heterogeneity. Breast Cancer Res.

[8] Lam SW, Jimenez CR, Boven E (2014). Breast cancer classification by proteomic technologies: current state of knowledge. Cancer Treat Rev.

[9] Perou CM, Sørlie T, Eisen MB, van de Rijn M, Jeffrey SS, Rees CA (2000). Molecular portraits of human breast tumours. Nature.

[10] Goldhirsch A, Wood WC, Coates AS, Gelber RD, Thürlimann B, Senn HJ (2011). Strategies for subtypes–dealing with the diversity of breast cancer: highlights of the St. Gallen International Expert Consensus on the primary therapy of early breast Cancer. Ann Oncol.

[11] Senkus E, Kyriakides S, Ohno S, Penault-Llorca F, Poortmans P, Rutgers E (2015). ESMO Guidelines Committee. Primary breast cancer: ESMO Clinical Practice Guidelines for diagnosis, treatment and follow-up. Ann Oncol.

[12] Sultan LR, Schultz SM, Cary TW, Sehgal CM. Machine learning to improve breast cancer diagnosis by multimodal ultrasound. IEEE Int Ultrason Symp. 2018; 2018:10. 10.1109/ultsym.2018.8579953 PMC8293293 34295453

[13] Itoh A, Ueno E, Tohno E, Kamma H, Takahashi H, Shiina T (2006). Breast disease: clinical application of US elastography for diagnosis. Radiology.

[14] Adler DD, Carson PL, Rubin JM, Quinn-Reid D (1990). Doppler ultrasound color flow imaging in the study of breast cancer: preliminary findings. Ultrasound Med Biol.

[15] Atri M, Hudson JM, Sinaei M, Williams R, Milot L, Moshonov H (2016). Impact of Acquisition Method and Region of Interest Placement on Inter-observer Agreement and Measurement of Tumor response to targeted therapy using dynamic contrast-enhanced Ultrasound. Ultrasound Med Biol.

[16] Feng Y, Qin XC, Luo Y, Li YZ, Zhou X (2015). Efficacy of contrast-enhanced ultrasound washout rate in predicting hepatocellular carcinoma differentiation. Ultrasound Med Biol.

[17] Wilson SR, Kim TK, Jang HJ, Burns PN (2007). Enhancement patterns of focal liver masses: discordance between contrast-enhanced sonography and contrast-enhanced CT and MRI. AJR Am J Roentgenol.

[18] Bhayana D, Kim TK, Jang HJ, Burns PN, Wilson SR (2010). Hypervascular liver masses on contrast-enhanced ultrasound: the importance of washout. AJR Am J Roentgenol.

[19] Lassau N, Bonastre J, Kind M, Vilgrain V, Lacroix J, Cuinet M (2014). Validation of dynamic contrast-enhanced ultrasound in predicting outcomes of antiangiogenic therapy for solid tumors: the french multicenter support for innovative and expensive techniques study. Invest Radiol.

[20] Cao X, Xue J, Zhao B (2012). Potential application value of contrast-enhanced ultrasound in neoadjuvant chemotherapy of breast cancer. Ultrasound Med Biol.

[21] Fröhlich E, Muller R, Cui XW, Schreiber-Dietrich D, Dietrich CF (2015). Dynamic contrast-enhanced ultrasound for quantification of tissue perfusion. J Ultrasound Med.

[22] Hudson JM, Williams R, Tremblay-Darveau C, Sheeran PS, Milot L, Bjarnason GA (2015). Dynamic contrast enhanced ultrasound for therapy monitoring. Eur J Radiol.

[23] Kim Y, Stolarska MA, Othmer HG (2011). The role of the microenvironment in tumor growth and invasion. Prog Biophys Mol Biol.

[24] Wu JS, Sheng SR, Liang XH, Tang YL (2017). The role of tumor microenvironment in collective tumor cell invasion. Future Oncol.

[25] Lee AK, DeLellis RA, Silverman ML, Heatley GJ, Wolfe HJ (1990). Prognostic significance of peritumoral lymphatic and blood vessel invasion in node-negative carcinoma of the breast. J Clin Oncol.

[26] Mohammed ZM, McMillan DC, Edwards J, Mallon E, Doughty JC, Orange C (2013). The relationship between lymphovascular invasion and angiogenesis, hormone receptors, cell proliferation and survival in patients with primary operable invasive ductal breast cancer. BMC Clin Pathol.

[27] Freed M, Storey P, Lewin AA, Babb J, Moccaldi M, Moy L (2016). Evaluation of breast lipid composition in patients with Benign tissue and Cancer by using multiple Gradient-Echo MR Imaging. Radiology.

[28] Shin HJ, Park JY, Shin KC, Kim HH, Cha JH, Chae EY (2016). Characterization of tumor and adjacent peritumoral stroma in patients with breast cancer using high-resolution diffusion-weighted imaging: correlation with pathologic biomarkers. Eur J Radiol.

[29] Cheon H, Kim HJ, Kim TH, Ryeom HK, Lee J, Kim GC (2018). Invasive breast Cancer: Prognostic Value of Peritumoral Edema identified at preoperative MR Imaging. Radiology.

[30] Barzaman K, Karami J, Zarei Z, Hosseinzadeh A, Kazemi MH, Moradi-Kalbolandi S (2020). Breast cancer: Biology, biomarkers, and treatments. Int Immunopharmacol.

[31] Masannat YA, Agrawal A, Maraqa L, Fuller M, Down SK, Tang S (2020). Multifocal and multicentric breast cancer, is it time to think again?. Ann R Coll Surg Engl.

[32] Chen X, Hu H, He L, Yu X, Liu X, Zhong R (2016). A novel subtype classification and risk of breast cancer by histone modification profiling. Breast Cancer Res Treat.

[33] Yu F, Quan F, Xu J, Zhang Y, Xie Y, Zhang J (2019). Breast cancer prognosis signature: linking risk stratification to disease subtypes. Brief Bioinform.

[34] Hayashi M, Yamamoto Y, Sueta A, Tomiguchi M, Yamamoto-Ibusuki M, Kawasoe T (2015). Associations between Elastography Findings and clinicopathological factors in breast Cancer. Med (Baltim).

[35] Yoo J, Seo BK, Park EK, Kwon M, Jeong H, Cho KR (2020). Tumor stiffness measured by shear wave elastography correlates with tumor hypoxia as well as histologic biomarkers in breast cancer. Cancer Imaging.

[36] Millar EK, Browne LH, Beretov J, Lee K, Lynch J, Swarbrick A et al. Tumour Stroma Ratio Assessment Using Digital Image Analysis Predicts Survival in Triple Negative and Luminal Breast Cancer. Cancers (Basel). 2020 Dec;12:3749. 10.3390/cancers12123749 PMC7764351 33322174

[37] Wu T, Li J, Wang D, Leng X, Zhang L, Li Z, Cho N, Jang M, Lyou CY, Park JS, Choi HY, Moon WK et al. Distinguishing benign from malignant masses at breast US: combined US elastography and color doppler US–influence on radiologist accuracy. Radiology. 2012; 262:80–90. 10.1148/radiol.11110886 22084209

[38] Cho N, Jang M, Lyou CY, Park JS, Choi HY, Moon WK (2012). Distinguishing benign from malignant masses at breast US: combined US elastography and color doppler US--influence on radiologist accuracy. Radiology.

[39] Ozdemir A, Ozdemir H, Maral I, Konuş O, Yücel S, Işik S (2001). Differential diagnosis of solid breast lesions: contribution of Doppler studies to mammography and gray scale imaging. J Ultrasound Med.

[40] Yu TF, He W, Gan CG, Zhao MC, Zhu Q, Zhang W (2021). Deep learning applied to two-dimensional color doppler flow imaging ultrasound images significantly improves diagnostic performance in the classification of breast masses: a multicenter study. Chin Med J (Engl).

[41] Angius A, Pira G, Cossu-Rocca P, Sotgiu G, Saderi L, Muroni MR et al. Deciphering clinical significance of BCL11A isoforms and protein expression roles in triple-negative breast cancer subtype.J Cancer Res Clin Oncol. 2022 Aug28. 10.1007/s00432-022-04301-w PMC10314865 36030436

[42] Bando Y, Kobayashi T, Miyakami Y, Sumida S, Kakimoto T, Saijo Y Triple-negative breast cancer and basal-like subtype†Pathology and targeted therapy. J Med Invest., Mandai M, Koda M, Matono T, Nagahara T, Sugihara T, Ueki M et al. Assessment of hepatocellular carcinoma by contrast-enhanced ultrasound with perfluorobutane microbubbles: comparison with dynamic CT. Br J Radiol. 2011; 84:499–507. 10.1259/bjr/38682601 PMC3473627 20959373

[43] AMandai M, Koda M, Matono T, Nagahara T, Sugihara T, Ueki M, et al. Assessment of hepatocellular carcinoma by contrast-enhanced ultrasound with perfluorobutane microbubbles: comparison with dynamic CT. Br J Radiol. 2011;84:499–507. 10.1259/bjr/38682601 PMC3473627 20959373

[44] Wang Y, Fan W, Zhao S, Zhang K, Zhang L, Zhang P (2016). Qualitative, quantitative and combination score systems in differential diagnosis of breast lesions by contrast-enhanced ultrasound. Eur J Radiol.

[45] Zhao YX, Liu S, Hu YB, Ge YY, Lv DM (2017). Diagnostic and prognostic values of contrast-enhanced ultrasound in breast cancer: a retrospective study. Onco Targets Ther.

[46] Jain RK (2005). Antiangiogenic therapy for Cancer: current and emerging concepts. Oncology.

[47] Thompson CB, Bauer DE, Lum JJ, Hatzivassiliou G, Zong WX, Zhao F (2005). How do cancer cells acquire the fuel needed to support cell growth?. Cold Spring Harb Symp Quant Biol.

[48] Sørlie T, Perou CM, Tibshirani R, Aas T, Geisler S, Johnsen H (2001). Gene expression patterns of breast carcinomas distinguish tumor subclasses with clinical implications. Proc Natl Acad Sci U S A.

[49] Specht JM, Kurland BF, Montgomery SK, Dunnwald LK, Doot RK, Gralow JR (2010). Tumor metabolism and blood flow as assessed by positron emission tomography varies by tumor subtype in locally advanced breast cancer. Clin Cancer Res.

